# Achieving Ultrastiff Polyampholyte Nanocomposite Hydrogels via the Synergistic Strategy of Effective Nanoparticle Aggregation and Multi-Bond Networks

**DOI:** 10.3390/gels12060523

**Published:** 2026-06-11

**Authors:** Mingzhen Wang, Shijun Long, Xuefeng Li, Yiwan Huang

**Affiliations:** 1 Hubei Provincial Key Laboratory of Green Materials for Light Industry, Hubei University of Technology, Wuhan 430068, China; 15343786501@163.com (M.W.);; 2 Hubei Longzhong Laboratory, Xiangyang 441000, China

**Keywords:** ultrastiff hydrogel, nanocomposite, metal-coordination bond, ionic bond, polyampholyte, synergistic reinforcement

## Abstract

Polyampholyte (PA) hydrogels have attracted considerable attention due to their unique dynamic network structures and favorable biocompatibility. However, their low modulus severely limits applications in load-bearing aspects. Herein, we report ultrastiff PA nanocomposite hydrogels through the synergistic strategy of effective aggregation of hydrophilic silica (SiO_2_) nanoparticles and multi-bond networks. Specifically, a high content of SiO_2_ nanoparticles is first incorporated into a dynamic ionic PA network *via in situ* polymerization. The resulting hydrogel is subsequently dialyzed in a zirconium salt solution with strong coordination capability, achieving the ultrastiff nanocomposite hydrogel. In this strategy, the dynamic PA network infiltrated between the aggregated SiO_2_ nanoparticles enables effective particle aggregation, while the dynamic PA network, consisting of ionic and metal-coordination bonds, provides efficient energy dissipation, resulting in a synergistic reinforcement effect. The effects of dialysis time, concentration of zirconium salt, and particle content on the swelling and mechanical behaviors of the hydrogels are systematically investigated. The optimized nanocomposite hydrogel exhibits a Young’s modulus and a tensile strength as high as 87.9 ± 5.9 MPa and 7.9 ± 0.1 MPa, respectively, which are 976 and 8.8 times those of the original neat PA hydrogel. This work provides an effective strategy for designing hydrogels with ultrahigh mechanical performance.

## 1. Introduction

Hydrogels have shown great potential in diverse fields such as tissue engineering [1,2,3,4,5] and flexible electronics [6,7,8,9,10] due to their high-water-content and soft tissue-like properties. However, conventional hydrogels usually suffer from poor mechanical performance, which limits their practical applications [11,12]. Significant efforts have been made to develop strong and tough hydrogels including double-network hydrogel, nanocomposite hydrogel, and dynamic network hydrogel [13,14,15,16,17,18]. For instance, polyampholyte (PA) hydrogels, synthesized by random copolymerization of positively and negatively charged monomers, feature extensive ionic bonds within the network that can act as dynamic sacrificial bonds to dissipate energy, thereby imparting high toughness and self-recovery characteristics [19,20,21,22]. Despite these merits, their modulus and strength are still relatively low, limiting their applications in load-bearing areas.

To enhance the mechanical properties of PA hydrogels, several strategies have been developed, including micro/nanocomposites [23,24] and multi-bond networks [25,26,27,28]. Metal-coordination bonds, owing to their reversibility and high bond energy, can significantly improve strength and modulus while maintaining toughness. In our recent work, we have reported a strategy for strengthening PA hydrogels by introducing multivalent metal ions (Zr^4+^) during dialysis, leveraging metal-coordination bonds to induce multiphase microstructural reorganization. In that hydrogel system, the introduced Zr^4+^ ions, which exist in solution as hydrophilic [Zr(OH)_2_·4H_2_O]_4_^8+^ clusters, exhibited strong coordination capability and could induce multiphase microstructural reorganization in PA hydrogels, resulting in an unusual “swelling-yet-strengthening” behavior (i.e., swelling in volume but strengthening in mechanical strength) [26,29,30,31,32]. The mechanical reinforcement of the hydrogels was mainly attributed to the synergy of ionic and metal-coordination bonds.

**Figure 1:**
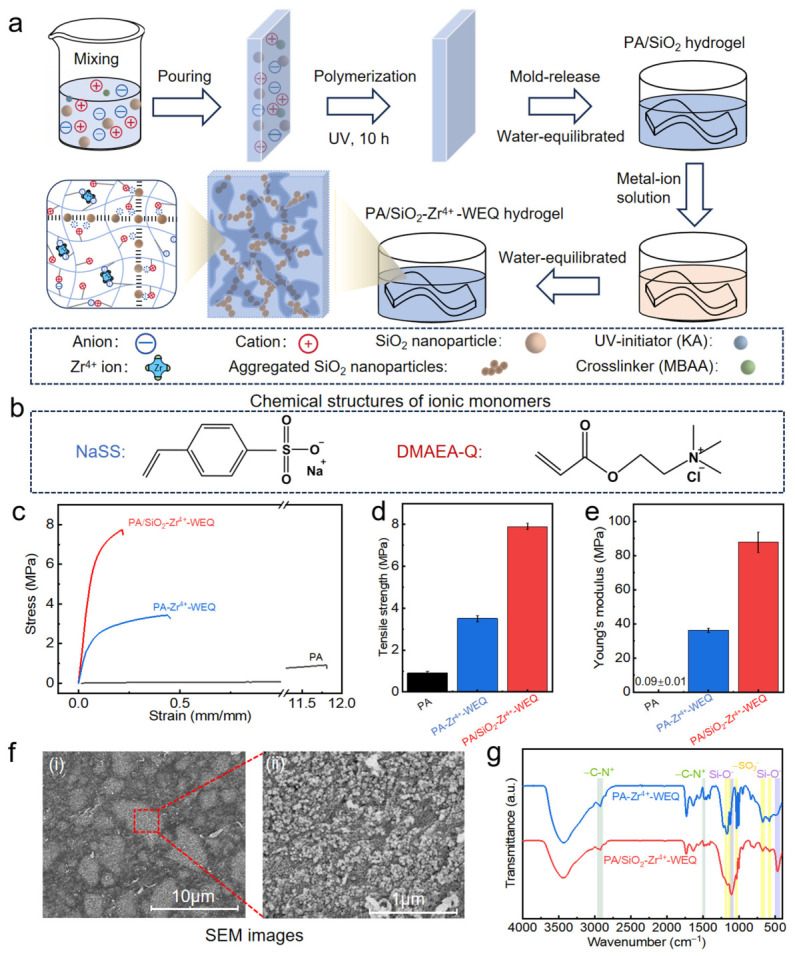
Design, fabrication, tensile properties, and structural characterizations of ultrastiff PA/SiO_2_-Zr^4+^ nanocomposite hydrogels. (**a**) Design strategy and fabrication process. (**b**) Chemical structures of ionic monomers used in this study. (**c**–**e**) Tensile behavior of neat PA hydrogel, PA-Zr^4+^-WEQ hydrogel, and PA/SiO_2_-Zr^4+^-WEQ hydrogel: stress–strain curves (**c**), tensile strength, *σ*_b_ (**d**), and Young’s modulus, *E* (**e**). (**f**) SEM images of PA/SiO_2_-Zr^4+^-WEQ hydrogel. Subfigure (**ii**) is the partial magnification of subfigure (**i**). (**g**) FTIR spectra of PA/SiO_2_-Zr^4+^-WEQ hydrogel. $C_{{\mathrm{ZrOCl}}_{2}}$ = 0.5 mol/L; *ω*_SiO2_ = 20 wt%.

In an extension of our recent work on the strengthening of PA hydrogels [26], here we design and fabricate ultrastiff PA-based nanocomposite hydrogels by synergizing effective aggregation of hydrophilic SiO_2_ nanoparticles and multi-bond network structure (Figure 1a). In detail, a high content of SiO_2_ nanoparticles is first embedded in a dynamic ionic PA network *via in situ* solution radical polymerization, achieving a PA/SiO_2_ hydrogel. The PA network is composed of a copolymer of P(NaSS-*co*-DMAEA-Q) from an anionic monomer [sodium *p*-styrenesulfonate (NaSS)] and a cationic monomer [dimethylaminoethylacrylate quaternized ammonium (DMAEA-Q)] (Figure 1b). The as-prepared PA/SiO_2_ hydrogel is then dialyzed in ZrOCl_2_ solution and deionized water in sequence, where the equilibration should be reached for each dialysis medium, obtaining the ultrastiff PA/SiO_2_-Zr^4+^ nanocomposite hydrogel. In our design, the effective aggregation of nanoparticles is achieved by *in situ* penetrated dynamic PA chains between the aggregated SiO_2_ nanoparticles during polymerization. The dynamic PA-based network, composed of a multi-bond (i.e., ionic and metal-coordination bonds) network, provides efficient energy dissipation, enabling synergistic mechanical enhancement. Systematic studies on the effects of dialysis time, ZrOCl_2_ concentration, and particle content on the swelling and mechanical properties are carried out. The effect of SiO_2_ nanoparticles on the chemical structure of the nanocomposite hydrogels is also discussed. After optimization, the Young’s modulus and tensile strength of our fabricated nanocomposite hydrogel reach 87.9 ± 5.9 MPa and 7.9 ± 0.1 MPa, respectively, which are 976 times and 8.8 times those of the neat PA hydrogel. Additionally, compared with our previous PA-Zr^4+^ hydrogels [26], our fabricated PA/SiO_2_-Zr^4+^ nanocomposite gel also shows significant increases in these mechanical parameters. This study provides a novel strategy for developing ultrastiff and strong nanocomposite hydrogels.

## 2. Results and Discussion

### 2.1. Design, Fabrication, and Concept Proof

Figure 1a illustrates the design principle and fabrication process of the PA/SiO_2_-Zr^4+^ hydrogels. Based on our strategy combining effective aggregation of SiO_2_ nanoparticles with multi-bond networks for synergistic reinforcement, a high content of SiO_2_ nanoparticles was first incorporated into the PA network, composed of dynamic ionic bonds, *via in situ* polymerization. The resulting hydrogels were subsequently dialyzed in a zirconium salt solution with strong coordination capability, ultimately achieving ultrastiff PA nanocomposite hydrogels. In this design and fabrication, the abundant cationic groups in the PA network interacted electrostatically with the negatively charged hydrophilic SiO_2_ nanoparticles, enabling effective aggregation of a high content of nanoparticles. Meanwhile, the metal-coordination bonds introduced through the secondary dialysis step could work synergistically with the existing ionic bonds in the PA network to optimize the hydrogel matrix, facilitating efficient energy dissipation and thereby achieving synergistic reinforcement.

For clarity, the as-prepared hydrogels after initial water equilibration are denoted as PA hydrogels; the composite hydrogels containing SiO_2_ nanoparticles are denoted as PA/SiO_2_ hydrogels; after the introduction of zirconium ions and subsequent secondary water equilibration, the gels are denoted as PA-Zr^4+^-WEQ or PA/SiO_2_-Zr^4+^-WEQ hydrogels, respectively.

To verify our design concept, representative samples were selected to evaluate their tensile properties, and the results are shown in Figure 1c–e. Here, $C_{{\mathrm{ZrOCl}}_{2}}$ is 0.5 mol/L and *ω*_SiO2_ is 20 wt%. The tensile data revealed that PA/SiO_2_-Zr^4+^-WEQ composite hydrogels prepared using our synergistic strategy exhibited a Young’s modulus and a tensile strength as high as 87.9 ± 5.9 MPa and 7.9 ± 0.1 MPa, respectively, which are 976 times and 8.8 times those of the original neat PA hydrogels, demonstrating a remarkable enhancement in mechanical properties. In addition, compared with the PA-Zr^4+^-WEQ hydrogel containing only multiple dynamic bonds, the composite hydrogel prepared in this work still showed significantly improved mechanical performance. These mechanical properties are notably superior to those of many reported high-strength and tough hydrogels [33,34,35,36].

The aggregation of SiO_2_ nanoparticles within the hydrogel matrix was clearly evidenced by the SEM images (Figure 1f). In addition, we also compared the chemical structures between the neat and composite hydrogels by FTIR spectra (Figure 1g). In the PA hydrogel network, the characteristic peaks of 1170 cm^−1^, 1123 cm^−1^, 1036 cm^−1^, 676 cm^−1^, and 583 cm^−1^ are correlated to –SO_3_^−^ groups, and the peaks of 2933 cm^−1^ and 1487 cm^−1^ are correlated to -C(CH_3_)_3_N^+^ (i.e., -C-N^+^) groups. The characteristic peaks of –SiO^−^ groups in SiO_2_ nanoparticles are located at 1102 cm^−1^ and 467 cm^−1^ (Figure S1). After the introduction of SiO_2_ nanoparticles, the characteristic peaks of the –SO_3_^−^ and -C(CH_3_)_3_N^+^ groups became weakened, but the peaks of –SiO^−^ groups from the SiO_2_ nanoparticles became stronger, indicating the existence of effective interactions (e.g., electrostatic attraction) between the nanoparticles and the hydrogel network. The effective interaction is important for the proposed “effective aggregation of SiO_2_ nanoparticles”. These combined results preliminarily confirmed our synergistic stiffening and strengthening strategy.

### 2.2. Effect of Dialysis Time in ZrOCl_2_ Solution

Our recent study revealed that, owing to the strong coordination capability of highly hydrated Zr^4+^ ion clusters and the multiphase structural characteristics of the neat PA hydrogel, the dialysis of the PA hydrogel in ZrOCl_2_ solution required several months to reach equilibrium, accompanied by increases in both size and mechanical properties [26]. In contrast, the subsequent re-equilibration in deionized water took only a few days (around 7 days). This process ultimately achieved a multiphase network structure reconstructed through the synergistic effect of ionic and metal-coordination bonds, exhibiting a swelling-yet-strengthening behavior. Therefore, in this section, we systematically investigate the effect of dialysis time on the swelling and mechanical behaviors of the fabricated nanocomposite hydrogels.

**Figure 2:**
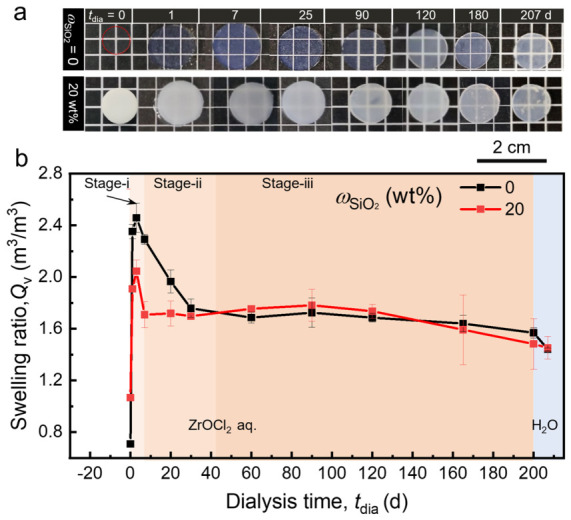
Swelling behavior of PA hydrogel and PA/SiO_2_-20 hydrogel in 0.5 mol/L ZrOCl_2_ solution and deionized water for different dialysis times (*t*_dia_). (**a**) Macroscopic photos of the samples. The red dotted circle around the sample in (**a**) are manually drawn as a visual guide. (**b**) Volume swelling ratio (*Q*_v_) versus *t*_dia_. Different background colors in (**b**) represent different swelling stages. The samples were subjected to secondary water equilibration after 200 d.

Figure 2 illustrates the swelling behavior of neat PA hydrogel and PA/SiO_2_-20 nanocomposite hydrogel after dialysis in 0.5 mol/L ZrOCl_2_ solution for different time, followed by secondary water equilibration. After the introduction of Zr^4+^ ions, the color change in PA-Zr^4+^ gels by the introduction of Zr^4+^ is mainly attributed to the reorganization of the multiphase microstructure of the PA network (Figure 2a), agreeing well with our recent study [26]. The color change in PA/SiO_2_-Zr^4+^ gels should be due to the combination effect between the structure color of PA-Zr^4+^ network and the surface reflection of aggregated SiO_2_ nanoparticles. The swelling plots reveal that the swelling process for both the neat and nanocomposite hydrogels can be divided into three stages (Figure 2b), in good agreement with our recent study on PA-Zr^4+^ hydrogels [26]. Stage I (0–3 d) is a rapid swelling stage, during which Zr^4+^ diffused into the gel network, disrupting the original ionic bonds and leading to a rapid increase in volume. Stage II (3–40 d) is a slow deswelling stage, where Zr^4+^ began to form metal-coordination bonds with the -SO_3_^−^ groups in the PA network, making the network gradually contract. Stage III (40–200 d) is a network reorganization and stabilization stage, during which the gel volume approached plateau values and the multiphase microstructure was essentially formed. After the secondary water equilibration of around 7 days, both the gel volumes showed clear increases compared to their initial states. This specific swelling behavior is consistent with recently reported data [26]. Compared with the neat PA hydrogel, the nanocomposite hydrogel exhibited a marked decrease in volume swelling ratio (*Q*_v_) during Stages I and II. However, the final swelling ratios of the two samples were comparable. The inorganic SiO_2_ nanoparticles are stable and cannot be compressed in volume in the hydrogel networks. In this case, it is reasonable that although the polymer network deswelled into a smaller volume for the nanocomposite hydrogel due to the effective interaction, the total volume change in the nanocomposite hydrogel could be comparable to the neat one. This result also suggests that the interaction between the effectively aggregated nanoparticles and the hydrogel network suppressed the swelling of the PA hydrogel network. This interaction probably benefits the mechanical enhancement of the nanocomposite hydrogels.

**Figure 3:**
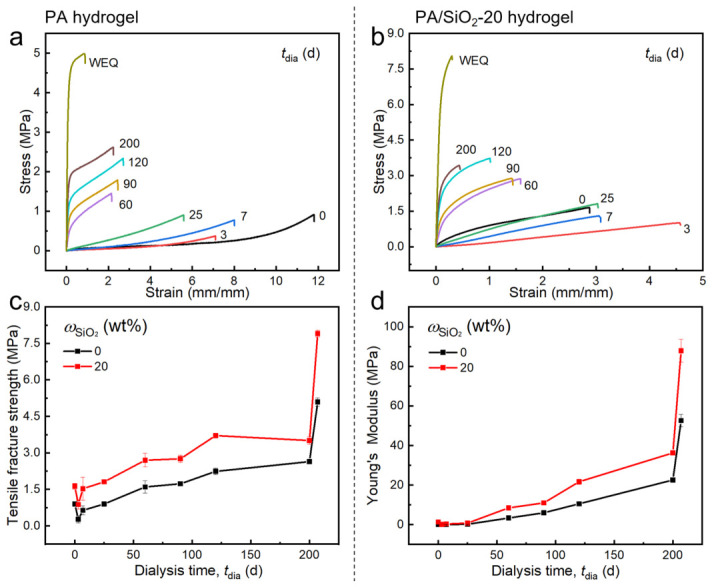
Tensile behaviors of PA hydrogel and PA/SiO_2_-20 nanocomposite hydrogel in 0.5 mol/L ZrOCl_2_ solution and deionized water for different *t*_dia_. (**a**,**b**) Stress–strain curves of the samples. (**c**,**d**) Detailed tensile properties of the samples.

We further investigated the effect of dialysis time (*t*_dia_) on the tensile properties of the hydrogels. Figure 3 presents the tensile stress–strain curves of neat PA hydrogels and PA/SiO_2_-20 nanocomposite hydrogels, along with the corresponding changes in tensile properties as a function of *t*_dia_. Detailed tensile data are also presented in Table 1. With increasing *t*_dia_ in ZrOCl_2_ solution, both the neat PA hydrogels and nanocomposite hydrogels exhibited a similar trend in mechanical properties. In the initial stage (0–3 d), the mechanical properties showed a certain degree of decline; with extending *t*_dia_ (3–40 d), they were gradually improved; upon further extension of *t*_dia_ (40–200 d), the mechanical properties were further enhanced. This trend agrees well with the three stages proposed to explain the swelling behavior of the hydrogels (Figure 2b). In Stage I, the substantial introduction of Zr^4+^ ions and their counter ions disrupted the original ionic network, resulting in slight decreases in mechanical properties. In Stage II, the gradual formation of metal-coordination bonds and ionic bonds led to progressive increases in mechanical properties. In Stage III, further reinforcement of metal-coordination bonds and ionic bonds as well as the network rearrangement and optimization contributed to additional enhancements in mechanical properties.

After transferring the above ZrOCl_2_-equilibrated hydrogels to deionized water for secondary equilibration, the mechanical properties of the hydrogels reach their maximum values. Compared with the neat PA gel, PA/SiO_2_ nanocomposite hydrogel exhibits improved tensile properties, although the enhancement effect is much lower than PA/SiO_2_-Zr^4+^-WEQ nanocomposite hydrogel. Meanwhile, compared to the PA-Zr^4+^-WEQ gel, the significantly higher mechanical properties of PA/SiO_2_-Zr^4+^-WEQ nanocomposite hydrogel are attributed to the synergistic reinforcement effect of the effective aggregation of SiO_2_ nanoparticles and the multi-bond network within the composite hydrogel. The above results demonstrate that the dialysis time in ZrOCl_2_ solution could effectively regulate the mechanical properties of the PA/SiO_2_-Zr^4+^-WEQ nanocomposite hydrogels.

### 2.3. Effect of Concentration of ZrOCl_2_ Solution

Our recent studies have indicated that the concentration of metal ions could affect both the quantity and quality of metal-coordination bonds, influencing the dissociation, reorganization, and optimization of the original ionic PA network, which could ultimately affect the strengthening of the hydrogels [37]. Therefore, we systematically investigated the effect of $C_{{\mathrm{ZrOCl}}_{2}}$ (0–1 mol/L) on the swelling and mechanical properties of the nanocomposite hydrogels in this section.

**Figure 4:**
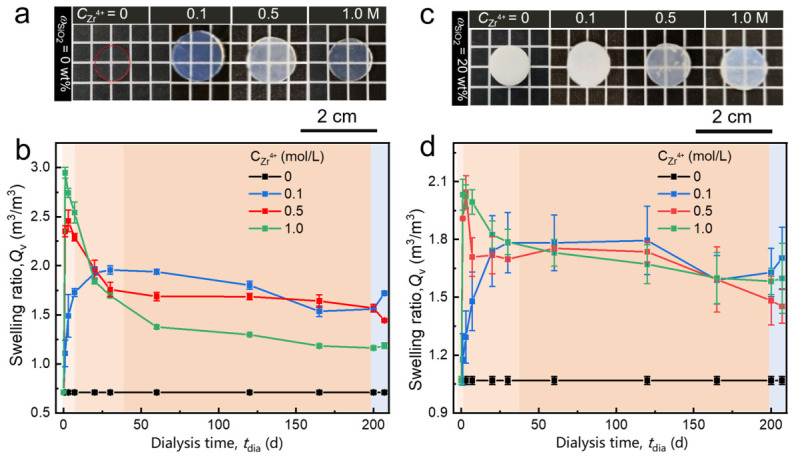
Swelling behavior of PA hydrogel (**a**,**b**) and PA/SiO_2_-20 hydrogel (**c**,**d**) in ZrOCl_2_ solutions with different $C_{{\mathrm{ZrOCl}}_{2}}$ and deionized water. (**a**,**c**) Macroscopic photos of the samples. The red dotted circle around the sample in (**a**) are manually drawn as a visual guide. (**b**,**d**) *Q*_v_ versus *t*_dia_ in dialysis solutions with different $C_{{\mathrm{ZrOCl}}_{2}}$. Different background colors in (**b**,**d**) represent different swelling stages similar to those shown in Figure 2b. The samples were subjected to secondary water equilibration after 200 d. Here $C_{{\mathrm{ZrOCl}}_{2}}$ = 0 mol/L in (**b**,**d**) means that the dialysis solution is deionized water.

We first studied the swelling behavior of neat PA hydrogels and PA/SiO_2_ nanocomposite hydrogels in ZrOCl_2_ solutions with different $C_{{\mathrm{ZrOCl}}_{2}}$, as presented in Figure 4. Compared with the ZrOCl_2_-un-dialyzed samples (i.e., $C_{{\mathrm{ZrOCl}}_{2}}$ = 0 mol/L), both the neat and composite hydrogels exhibited the aforementioned three characteristic swelling stages (Stage I: pronounced swelling; Stage II: slow deswelling; Stage III: further deswelling). Meanwhile, both kinds of hydrogels showed obvious swelling behaviors, which were markedly influenced by $C_{{\mathrm{ZrOCl}}_{2}}$. These swelling differences were primarily attributed to the fact that $C_{{\mathrm{ZrOCl}}_{2}}$ could determine the magnitude of the osmotic pressure difference, which accordingly affected the rate of zirconium ion diffusion into the PA network and the progress of reconstruction of the multi-bond-based dynamic network.

Specifically, with increasing $C_{{\mathrm{ZrOCl}}_{2}}$, the neat PA hydrogel samples exhibited more pronounced swelling in Stage I and showed a lower volume swelling ratio (*Q*_v_) after secondary water equilibration (Figure 4a,b). For the PA/SiO_2_ nanocomposite hydrogels, the swelling behavior in Stage I followed a similar trend to that of neat PA hydrogels with increasing $C_{{\mathrm{ZrOCl}}_{2}}$ (Figure 4c,d). However, the composite hydrogels achieved the lowest *Q*_v_ at $C_{{\mathrm{ZrOCl}}_{2}}$ = 0.5 mol/L. Additionally, PA/SiO_2_-Zr^4+^-WEQ nanocomposite hydrogels exhibited lower *Q*_v_s than PA/-Zr^4+^-WEQ hydrogels. This result indicates that the effective interactions between the SiO_2_ nanoparticles and the PA network suppressed the swelling of the composite network. Furthermore, only at an appropriate $C_{{\mathrm{ZrOCl}}_{2}}$ could the effective aggregation of nanoparticles and the multi-bond network effectively constrain network swelling, which should be beneficial for the mechanical enhancement of the nanocomposite hydrogels.

**Figure 5:**
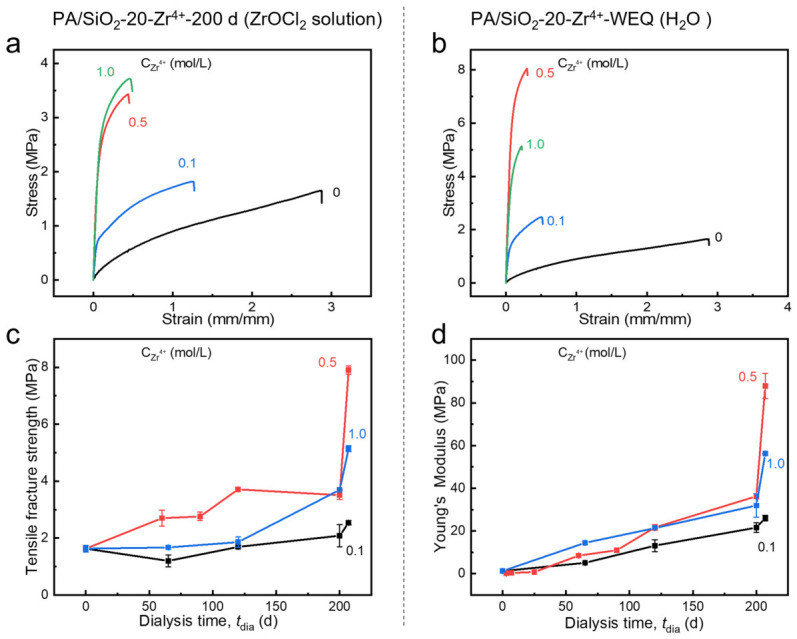
Tensile behaviors of PA/SiO_2_-Zr^4+^ hydrogels equilibrated in different ZrOCl_2_ solutions after 200 d and re-equilibrated in deionized water. (**a**,**b**) Stress–strain curves of the samples. (**c**,**d**) Detailed tensile properties of the samples. *ω*_SiO2_ = 20 wt%.

Next, we studied the effect of $C_{{\mathrm{ZrOCl}}_{2}}$ on the tensile behavior of PA/SiO_2_-Zr^4+^ nanocomposite hydrogels for the samples equilibrated in ZrOCl_2_ solutions and re-equilibrated in deionized water (Figure 5). The detailed tensile data are also presented in Table 2. It can be seen that most of the composite hydrogels exhibited enhanced mechanical properties, which increased continuously as $C_{{\mathrm{ZrOCl}}_{2}}$ increased. At $C_{{\mathrm{ZrOCl}}_{2}}$ = 0.1 mol/L, the enhanced mechanical properties of the composite hydrogels primarily resulted from the effective aggregation of SiO_2_ nanoparticles, because our previous study revealed that the contribution from the multi-bond network was very limited for this $C_{{\mathrm{ZrOCl}}_{2}}$ [26]. When dialyzed only in ZrOCl_2_ solutions, PA/SiO_2_-Zr^4+^ composite hydrogels exhibited the highest mechanical properties at $C_{{\mathrm{ZrOCl}}_{2}}$ = 1.0 mol/L. However, after the secondary water equilibration, the mechanical properties of PA/SiO_2_-Zr^4+^-WEQ composite hydrogels were optimized at $C_{{\mathrm{ZrOCl}}_{2}}$ = 0.5 mol/L. This difference is mainly attributed to the following: (i) excess Zr^4+^ ions might be not beneficial to the mechanical reinforcement of the PA network constructed *via* multi-bond interactions; (ii) excess Zr^4+^ ions and the counter ions might also reduce the interactions between the SiO_2_ nanoparticles and the polymer network due to charge screening effect, thereby compromising the synergistic reinforcement effect. Nevertheless, when $C_{{\mathrm{ZrOCl}}_{2}}$ was even as low as 0.1 mol/L, the Young’s modulus (*E*) and tensile strength (*σ*_b_) of PA/SiO_2_-Zr^4+^-WEQ composite hydrogel reached 26.1 MPa and 2.54 MPa, respectively, which are 290 times and 2.8 times those of original neat PA hydrogel. At $C_{{\mathrm{ZrOCl}}_{2}}$ = 0.5 mol/L, the *E* and *σ*_b_ of PA/SiO_2_-Zr^4+^-WEQ composite hydrogel reached 87.9 ± 5.9 MPa and 7.9 ± 0.1 MPa, respectively, which are 976 times and 8.8 times those of original neat one. Additionally, the tensile properties of PA/SiO_2_-Zr^4+^-WEQ composite hydrogels with different $C_{{\mathrm{ZrOCl}}_{2}}$ clearly surpassed those of PA-Zr^4+^-WEQ hydrogels (Figure S2), confirming again the synergistic reinforcement combining effective aggregation of SiO_2_ nanoparticles with multi-bond networks. The above results demonstrate that $C_{{\mathrm{ZrOCl}}_{2}}$ could effectively regulate the mechanical properties of the nanocomposite hydrogels.

### 2.4. Effect of Content of SiO_2_ Nanoparticles

The content of SiO_2_ nanoparticles (*ω*_SiO2_) should influence their distribution within the PA hydrogel matrix, accordingly affecting the efficiency of the synergistic reinforcement from the effective aggregation of nanoparticles and the multi-bond network proposed in our design strategy. In this section, we systematically discussed the effect of *ω*_SiO2_ on the swelling and mechanical properties of the fabricated PA/SiO_2_-Zr^4+^ nanocomposite hydrogels.

**Figure 6:**
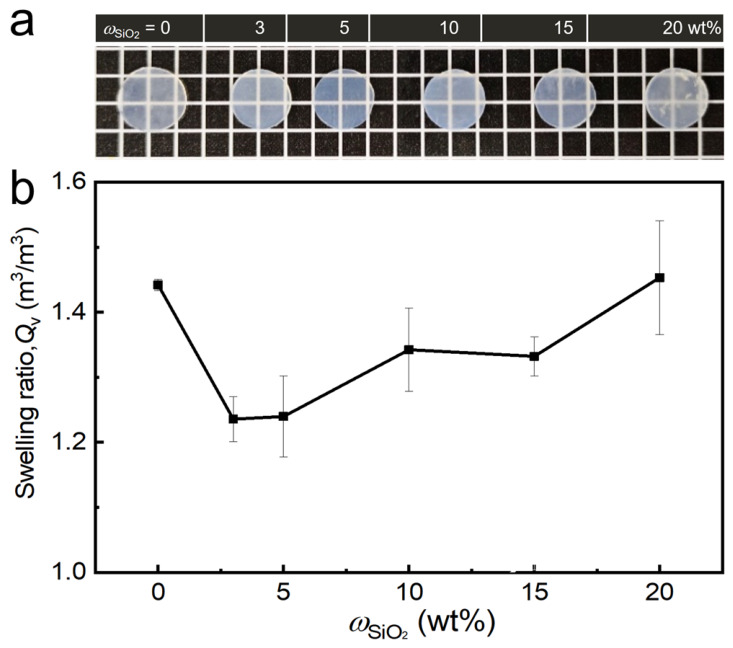
Swelling behavior of PA/SiO_2_-Zr^4+^-WEQ nanocomposite hydrogels with different *ω*_SiO2_. (**a**) Macroscopic photos of the samples. (**b**) *Q*_v_ versus *ω*_SiO2_. $C_{{\mathrm{ZrOCl}}_{2}}$ = 0.5 mol/L.

We first investigated the volume swelling ratio (*Q*_v_) of PA/SiO_2_-Zr^4+^-WEQ nanocomposite hydrogels after secondary water equilibration, as shown in Figure 6. Meanwhile, *Q*_v_ versus *t*_dia_ plots for the composite hydrogels with different *ω*_SiO2_ and $C_{{\mathrm{ZrOCl}}_{2}}$ (Figures S3–S5) indicate similar swelling processes with that shown in Figure 2. Clearly, the hydrogel without SiO_2_ nanoparticles (i.e., *ω*_SiO2_ = 0 wt%) exhibited the largest *Q*_v_. As *ω*_SiO2_ increased, the *Q*_v_ of the composite hydrogels initially became markedly lower than that of the original neat PA hydrogel, followed by a gradual increase. At *ω*_SiO2_ = 20 wt%, the composite hydrogel possessed a comparable *Q*_v_ with the neat gel. At relatively low *ω*_SiO2_ (0–10 wt%), the lower *Q*_v_s of the composite hydrogels were primarily attributed to the effective interactions between the SiO_2_ nanoparticles and the polymer network. At relatively high *ω*_SiO2_ (10–20 wt%), the higher *Q*_v_s of the composite hydrogels were mainly due to the incompressibility of the extensively aggregated SiO_2_ nanoparticles, limiting the volume contraction during the secondary water equilibration process. The effective interactions between the SiO_2_ nanoparticles and the polymer network served as the driving force for the “effective aggregation of nanoparticles” proposed in our design strategy.

**Figure 7:**
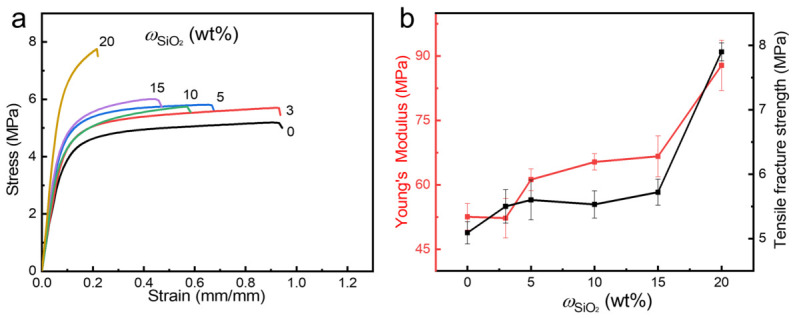
Tensile behaviors of PA/SiO_2_-Zr^4+^-WEQ nanocomposite hydrogels with different *ω*_SiO2_. (**a**) Stress–strain curves of the samples. (**b**) Detailed tensile properties of the samples. $C_{{\mathrm{ZrOCl}}_{2}}$ = 0.5 mol/L.

We further discussed the effect of *ω*_SiO2_ on the tensile properties of PA/SiO_2_-Zr^4+^-WEQ nanocomposite hydrogels with $C_{{\mathrm{ZrOCl}}_{2}}$ = 0.5 mol/L (Figure 7). The detailed tensile data are also presented in Table 3. It can be seen that, with increasing *ω*_SiO2_ (0–15 wt%), the Young’s modulus and tensile strength of the composite hydrogels exhibited gradual increases. Tensile data versus *t*_dia_ plots for the composite hydrogels with different *ω*_SiO2_ ($C_{{\mathrm{ZrOCl}}_{2}}$ = 0.5 mol/L) further indicate the dynamically adjusted tensile properties with *t*_dia_ due to the re-organized network structures (Figure S6), similar to the data shown in Figure 3. At *ω*_SiO2_ = 20 wt%, these tensile properties showed a significant enhancement. This behavior was primarily attributed to the dispersion state of the SiO_2_ nanoparticles within the hydrogel matrix. At relatively low *ω*_SiO2_, the nanoparticles were relatively uniformly dispersed, resulting in a moderate improvement in mechanical properties. In contrast, at relatively high *ω*_SiO2_, the effective aggregation of nanoparticles synergistically interacted with the dynamic multi-bond network, leading to a substantial mechanical enhancement of the composite hydrogels. The trend of tensile data against *ω*_SiO2_ for the composite hydrogels with different $C_{{\mathrm{ZrOCl}}_{2}}$ is slightly different, probably owing to the difference in energy dissipation efficiency of the multi-bond networks (Figures S6 and S7). Notably, the Young’s modulus of the composite hydrogel with *ω*_SiO2_ = 20 wt% increased by approximately three orders of magnitude compared to that of the original neat PA hydrogel. These tensile data further validated our design strategy.

**Figure 8:**
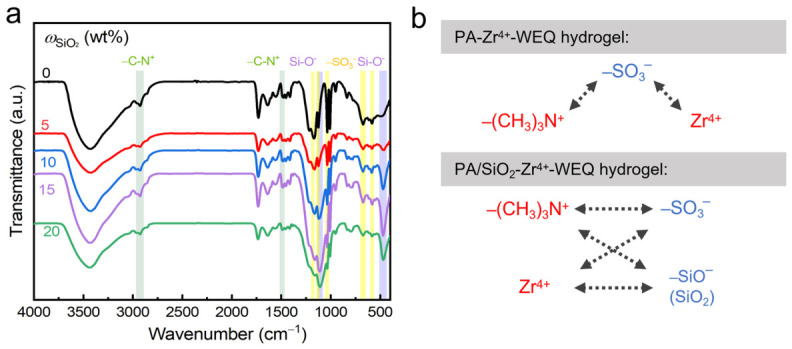
Chemical structures of PA/SiO_2_-Zr^4+^-WEQ nanocomposite hydrogels with different *ω*_SiO2_. (**a**) FTIR spectra of the samples. (**b**) Schematic illustration of the potential interactions in PA-Zr^4+^-WEQ hydrogel and PA/SiO_2_-Zr^4+^-WEQ hydrogel. Blue color represents negatively charged groups, and red color represents positively charged groups. Dashed double arrows indicate possible electrostatic attractions.

At last, we discussed the effect of *ω*_SiO2_ on the chemical structures of PA/SiO_2_-Zr^4+^-WEQ nanocomposite hydrogels by FTIR spectra (Figure 8a). It can be seen that, with increasing *ω*_SiO2_, the characteristic peaks of the –SO_3_^−^ and -C(CH_3_)_3_N^+^ groups in the PA network became gradually weakened, whereas the peaks of –SiO^−^ groups from the SiO_2_ nanoparticles became gradually stronger. This trend further indicates that the *in situ* introduced SiO_2_ nanoparticles could form effective interactions (e.g., electrostatic attraction) with PA-Zr^4+^ hydrogel matrix. We further illustrate the possible electrostatic interactions in both PA-Zr^4+^-WEQ hydrogel and PA/SiO_2_-Zr^4+^-WEQ nanocomposite hydrogel, as presented in Figure 8b. It is clearly seen that more possible interactions were introduced by addition of hydrophilic SiO_2_ nanoparticles in the nanocomposite hydrogel system. This result demonstrates that the incorporated SiO_2_ nanoparticles could effectively interact with the hydrogel matrix. The effective interactions are critical for achieving the “effective aggregation of nanoparticles” proposed in our design strategy, thereby ensuring the synergistic effect with the multi-bond network in enhancing the nanocomposite hydrogels.

## 3. Conclusions

In this work, we have successfully developed ultrastiff PA/SiO_2_-Zr^4+^ nanocomposite hydrogels *via* the synergistic strategy of effective aggregation of SiO_2_ nanoparticles and multi-bond networks. To achieve the nanocomposite hydrogels, a high content of SiO_2_ nanoparticles should be first introduced into a dynamic ionic PA network *via in situ* polymerization, followed by a dialysis in a ZrOCl_2_ solution. The dynamic PA network infiltrated between the aggregated SiO_2_ nanoparticles ensured the effective particle aggregation, which accordingly cooperated with the multi-bond (i.e., ionic and metal-coordination bonds) networks to enable the synergistic reinforcement. We systematically investigated the effects of dialysis time (*t*_dia_), ZrOCl_2_ concentration ($C_{{\mathrm{ZrOCl}}_{2}}$), and SiO_2_ content (*ω*_SiO2_) on the swelling and mechanical properties of the composite hydrogels. The effective aggregation of SiO_2_ nanoparticles in the PA network was confirmed by FTIR spectra. When *t*_dia_ = 200 d, $C_{{\mathrm{ZrOCl}}_{2}}$ = 0.5 mol/L, and *ω*_SiO2_ = 20 wt%, the nanocomposite hydrogel possesses the optimized tensile properties—a Young’s modulus of 87.9 ± 5.9 MPa and a tensile strength of 7.9 ± 0.1 MPa, which are 976 and 8.8 times those of the neat PA gel. The underlying strengthening mechanism and scale-up of PA/SiO_2_-Zr^4+^ nanocomposite hydrogels need to be further explored in future work. This study offers an effective strategy for developing ultrastiff hydrogels.

## 4. Materials and Methods

### 4.1. Materials

Sodium *p*-styrene sulfonate (NaSS, 90 wt%; anionic monomer) was purchased from Shanghai Aladdin Biochemical Technology Co., Ltd. (Shanghai, China) Methyl chloride quaternized *N*,*N*-dimethylamino ethylacrylate (DMAEA-Q, 80 wt%; cationic monomer) was obtained from J&K Chemical Ltd. (Beijing, China). [2-(methacryloyloxy)ethyl]trimethylammonium chloride (MPTC, 50 wt%; cationic monomer), *N,N*’-methylene-bis-acrylamide (MBAA, chemical crosslinker) were supplied by Sinopharm Chemical Reagent Co., Ltd. (Shanghai, China). *α*-ketoglutaric acid (*α*-keto; UV photo-initiator), ZrOCl_2_ and SiO_2_ nanoparticles (20 nm) were acquired from Shanghai Macklin Biochemical Technology Co., Ltd. (Shanghai, China).

### 4.2. Preparation of PA-SiO_2_ Hydrogels

PA/SiO_2_ hydrogels were prepared *via* free radical polymerization. The ionic monomers NaSS and DMAEA-Q (total monomer concentration *C*_m_ = 2.5 mol/L), crosslinker MBAA (0.10 mol%), and initiator KA (0.10 mol%) were dissolved in deionized water. The molar ratio of anionic NaSS and cationic DMAEA-Q was 0.51:0.49. Various mass fractions (0–20 wt%) of SiO_2_ nanoparticles were added, and the mixture was stirred in a 65 °C water bath for 15 min. The precursor solution was injected into a glass mold and irradiated with a UV lamp (365 nm, 4 W/cm^2^) for 10 h to obtain the as-prepared PA/SiO_2_ (ASP-PA/SiO_2_) gels. The gels were then dialyzed in deionized water for 5 days, with the water changed every 12 h, to obtain the water-equilibrated PA/SiO_2_ gels.

### 4.3. Preparation of PA/SiO_2_-Zr^4+^ Hydrogels

The PA/SiO_2_ gels were immersed in ZrOCl_2_ solutions at different concentrations (0.1 mol/L, 0.5 mol/L, 1.0 mol/L) for various durations (3–200 d). After 200 d, the gels were re-immersed in deionized water for 5 d to obtain the secondarily water-equilibrated PA/SiO_2_-Zr^4+^-WEQ hydrogels.

### 4.4. Swelling Ratio of Hydrogels

The as-prepared (ASP) cylindrical hydrogel samples were immersed in deionized (DI) water to attain a secondary re-equilibrium. The original sample volume *V*_0_ was determined based on the initial geometry (diameter *d*_0_ = 10 mm × thickness *t*_0_ ≈ 1 mm), while the volume *V*s at different immersion time were calculated from the measured dimensions (*d* × *t*). The volume swelling ratio (*Q*_v_) of the sample is defined as [26]

(1) $$Qv=\frac{V}{V_{0}}=\frac{d^{2}\cdot t}{{d_{0}}^{2}\cdot t_{0}}$$

### 4.5. Tensile Tests

Uniaxial tensile tests of hydrogel samples were performed on a universal testing machine (MTS, E43.104) equipped with a 250 N load cell. Prior to testing, the samples were cut into a dumbbell shape with dimensions of length [*l*] = 12 mm × width [*w*] = 2.5 mm × thickness [*t*] ≈ 1 mm. Tests were conducted at room temperature with a crosshead speed of 100 mm·min^−1^. The detailed sample geometry and method for the tests are illustrated in Figure S9. To prevent dehydration of the hydrogels, a humidifier was used during testing. The Young’s modulus (*E*) was calculated from the initial slope of the stress–strain curve within the 10% strain range. The work of tension during testing, denoted as *W*_b_, was determined by integrating the area under the stress–strain curve.

(2) $$W_{b}=\int_{0}^{\varepsilon_{b}}\sigma d\varepsilon$$

where *σ* and *ε* represent the stress and strain of the sample, respectively, and *ε*_b_ denotes the strain at fracture. Each sample was tested at least three times, and the average value was calculated and the standard deviation was obtained as error bar.

### 4.6. Fourier Transform Infrared Spectroscopy (FTIR)

FTIR spectral analysis of the hydrogel samples was performed on a Tensor 2 FTIR spectrometer (NeXus, Houston, TX, USA). Sample preparation involved the standard KBr pellet method, with spectral data acquired across the 400–4000 cm^−1^ range at 4 cm^−1^ resolution. All measurements were conducted under ambient temperature conditions.

Other experimental details are presented in the Supplementary Materials.

## Figures and Tables

**Table 1:** Summary of volume swelling ratio (*Q*_v_) and tensile properties of neat PA hydrogel, PA-Zr^4+^ hydrogels, and PA/SiO_2_-Zr^4+^ nanocomposite hydrogels with different *t*_dia_ and *ω*_SiO2_. $C_{{\mathrm{ZrOCl}}_{2}}$ = 0.5 mol/L.

Sample Code [x, y] ^(a)^	Young’s Modulus, *E* [MPa]	Tensile Strength, *σ*_b_ [MPa]	Work of Tension, *W*_b_ [MJ/m^3^]	*Q*_v_ [m^3^/m^3^]
PA	0.09 ± 0.01	0.90 ± 0.10	2.87 ± 0.17	0.71 ± 0.01
PA-Zr^4+^-3	0.04 ± 0.01	0.27 ± 0.15	0.64 ± 0.33	2.46 ± 0.11
PA-Zr^4+^-7	0.04 ± 0.02	0.64 ± 0.19	1.42 ± 0.22	2.29 ± 0.04
PA-Zr^4+^-25	0.17 ± 0.02	0.90 ± 0.10	2.28 ± 0.24	1.97 ± 0.09
PA-Zr^4+^-60	3.33 ± 0.07	1.60 ± 0.25	3.02 ± 0.95	1.69 ± 0.04
PA-Zr^4+^-90	6.00 ± 0.10	1.73 ± 0.07	3.10 ± 0.28	1.72 ± 0.11
PA-Zr^4+^-120	10.5 ± 0.1	2.24 ± 0.13	4.43 ± 0.60	1.68 ± 0.03
PA-Zr^4+^-200	22.6 ± 0.7	2.64 ± 0.03	4.66 ± 0.37	1.56 ± 0.40
PA-Zr^4+^-WEQ	52.6 ± 3.1	5.09 ± 0.17	4.20 ± 0.28	1.44 ± 0.01
PA/SiO_2_-20	1.24 ± 0.13	1.63 ± 0.12	2.65 ± 0.27	1.07 ± 0.02
PA/SiO_2_-20-Zr^4+^-3	0.14 ± 0.01	0.88 ± 0.13	1.75 ± 0.63	2.05 ± 0.09
PA/SiO_2_-20-Zr^4+^-7	0.37 ± 0.01	1.53 ± 0.47	1.49 ± 0.83	1.71 ± 0.10
PA/SiO_2_-20-Zr^4+^-25	0.75 ± 0.01	1.81 ± 0.15	3.05 ± 0.63	1.72 ± 0.10
PA/SiO_2_-20-Zr^4+^-60	8.48 ± 0.04	2.70 ± 0.28	2.41 ± 0.53	1.75 ± 0.01
PA/SiO_2_-20-Zr^4+^-90	11.0 ± 0.1	2.76 ± 0.15	2.60 ± 0.60	1.78 ± 0.12
PA/SiO_2_-20-Zr^4+^-120	21.6 ± 1.3	3.71 ± 0.01	3.04 ± 0.58	1.73 ± 0.54
PA/SiO_2_-20-Zr^4+^-200	36.3 ± 1.2	3.51 ± 0.15	1.15 ± 0.31	1.48 ± 0.19
PA/SiO_2_-20-Zr^4+^-WEQ	87.9 ± 5.9	7.90 ± 0.10	1.60 ± 0.70	1.45 ± 0.09

^(a)^ *x* and *y* in the code of PA/SiO_2_-*x*-Zr^4+^-*y* represent *ω*_SiO2_ (wt%) and *t*_dia_ (d), respectively.

**Table 2:** Summary of tensile properties of PA/SiO_2_ hydrogel and PA/SiO_2_-Zr^4+^ nanocomposite hydrogels with different $C_{{\mathrm{ZrOCl}}_{2}}$.

Sample Code [*x*, *y*, *z*] ^(a)^	*E* [MPa]	*σ*_b_ [MPa]	*W*_b_ [MJ/m^3^]
PA/SiO_2_-20	1.24 ± 0.13	1.63 ± 0.12	2.65 ± 0.27
PA/SiO_2_-20-0.1-Zr^4+^-200	21.6 ± 2.2	2.09 ± 0.38	1.21 ± 0.41
PA/SiO_2_-20-0.5-Zr^4+^-200	36.3 ± 1.2	3.51 ± 0.15	1.15 ± 0.31
PA/SiO_2_-20-1.0-Zr^4+^-200	31.9 ± 2.5	3.70 ± 0.02	1.35 ± 0.21
PA/SiO_2_-20-0.1-Zr^4+^-WEQ	26.1 ± 1.3	2.54 ± 0.08	1.23 ± 0.33
PA/SiO_2_-20-0.5-Zr^4+^-WEQ	87.9 ± 5.9	7.90 ± 0.10	1.60 ± 0.70
PA/SiO_2_-20-1.0-Zr^4+^-WEQ	56.2 ± 0.1	5.14 ± 0.10	0.86 ± 0.10

^(a)^ *x*, *y*, and *z* in the code of PA/SiO_2_-*x*-*z*-Zr^4+^-*y* represent *ω*_SiO2_ (wt%), *t*_dia_ (d), and $C_{{\mathrm{ZrOCl}}_{2}}$ (mol/L), respectively.

**Table 3:** Summary of tensile properties of PA/SiO_2_-Zr^4+^-WEQ nanocomposite hydrogels with different *ω*_SiO2_. $C_{{\mathrm{ZrOCl}}_{2}}$ = 0.5 mol/L.

Sample Code [*x*] ^(a)^	*E* [MPa]	*σ*_b_ [MPa]	*W*_b_ [MJ/m^3^]
PA-Zr^4+^-WEQ	52.6 ± 3.1	5.09 ± 0.17	4.20 ± 0.28
PA/SiO_2_-3-Zr^4+^-WEQ	52.3 ± 4.6	5.50 ± 0.26	3.27 ± 0.25
PA/SiO_2_-5-Zr^4+^-WEQ	61.2 ± 5.5	5.60 ± 0.31	5.30 ± 0.85
PA/SiO_2_-10-Zr^4+^-WEQ	65.4 ± 1.9	5.72 ± 0.20	1.90 ± 0.55
PA/SiO_2_-15-Zr^4+^-WEQ	66.7 ± 4.8	5.53 ± 0.21	2.40 ± 0.56
PA/SiO_2_-20-Zr^4+^-WEQ	87.9 ± 5.9	7.90 ± 0.14	1.60 ± 0.65

^(a)^ *x* in the code of PA/SiO_2_-*x*-Zr^4+^-WEQ represents *ω*_SiO2_ (wt%).

## Data Availability

The data that support the findings of this study are available from the corresponding author upon reasonable request.

## Supplementary Materials

The following supporting information can be downloaded at https://www.mdpi.com/article/10.3390/gels12060523/s1, Figure S1: FTIR spectrum of SiO_2_ nanoparticles used in this study. Figure S2: Tensile behaviors of PA-Zr^4+^ hydrogels equilibrated in different ZrOCl_2_ solutions after 200 d and re-equilibrated in deionized water. Figure S3: Swelling behavior of PA/SiO_2_ nanocomposite hydrogel in 0.5 mol/L ZrOCl_2_ solution and deionized water for different dialysis time (*t*_dia_). Figure S4: Swelling behavior of PA/SiO_2_ nanocomposite hydrogel in 0.1 mol/L ZrOCl_2_ solution and deionized water for different dialysis time (*t*_dia_). Figure S5: Swelling behavior of PA/SiO_2_ nanocomposite hydrogel in 1.0 mol/L ZrOCl_2_ solution and deionized water for different dialysis time (*t*_dia_). Figure S6: Tensile properties of PA/SiO_2_ nanocomposite hydrogel in 0.5 mol/L ZrOCl_2_ solution and deionized water for different dialysis time (*t*_dia_). Figure S7: Tensile properties of PA/SiO_2_ nanocomposite hydrogel in 0.1 mol/L ZrOCl_2_ solution and deionized water for different dialysis time (*t*_dia_). Figure S8: Tensile properties of PA/SiO_2_ nanocomposite hydrogel in 1.0 mol/L ZrOCl_2_ solution and deionized water for different dialysis time (*t*_dia_). Figure S9: Sample geometry and method for tensile tests.
