# Supplementary material for: Achieving Ultrastiff Polyampholyte Nanocomposite Hydrogels via the Synergistic Strategy of Effective Nanoparticle Aggregation and Multi-Bond Networks
Source: Gels. 2026 Jun 11;12(6):523. doi: 10.3390/gels12060523 (PMC13298286; doi:10.3390/gels12060523)
Supplement: Supplementary file 1 [file gels-12-00523-s001.zip › gels-4268191-supplementary.pdf]

Supplementary Materials

Achieving Ultrastiff Polyampholyte Nanocomposite Hydrogels via the Synergistic Strategy of Effective Nanoparticle Aggregation and Multi-Bond Networks

Mingzhen Wang [1], Shijun Long [1], Xuefeng Li [1] and Yiwan Huang [1,2,*]

[1] Hubei Provincial Key Laboratory of Green Materials for Light Industry, Hubei University of Technology, Wuhan 430068, China; 15343786501@163.com (M.W.)
[2] Hubei Longzhong Laboratory, Xiangyang 441000, China
* Correspondence: yiwanhuang@hbut.edu.cn

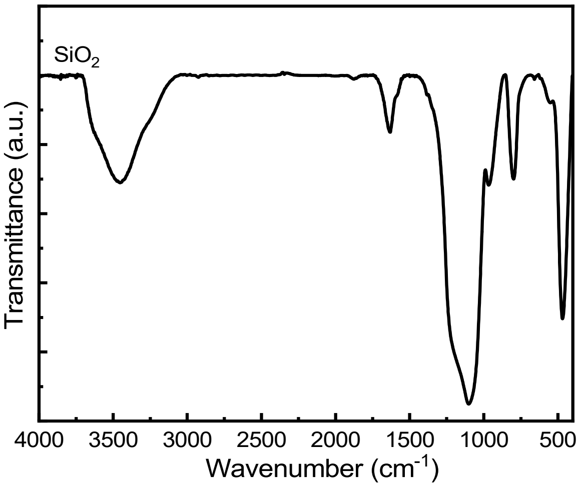

Figure S1. FTIR spectrum of SiO_2 nanoparticles used in this study.

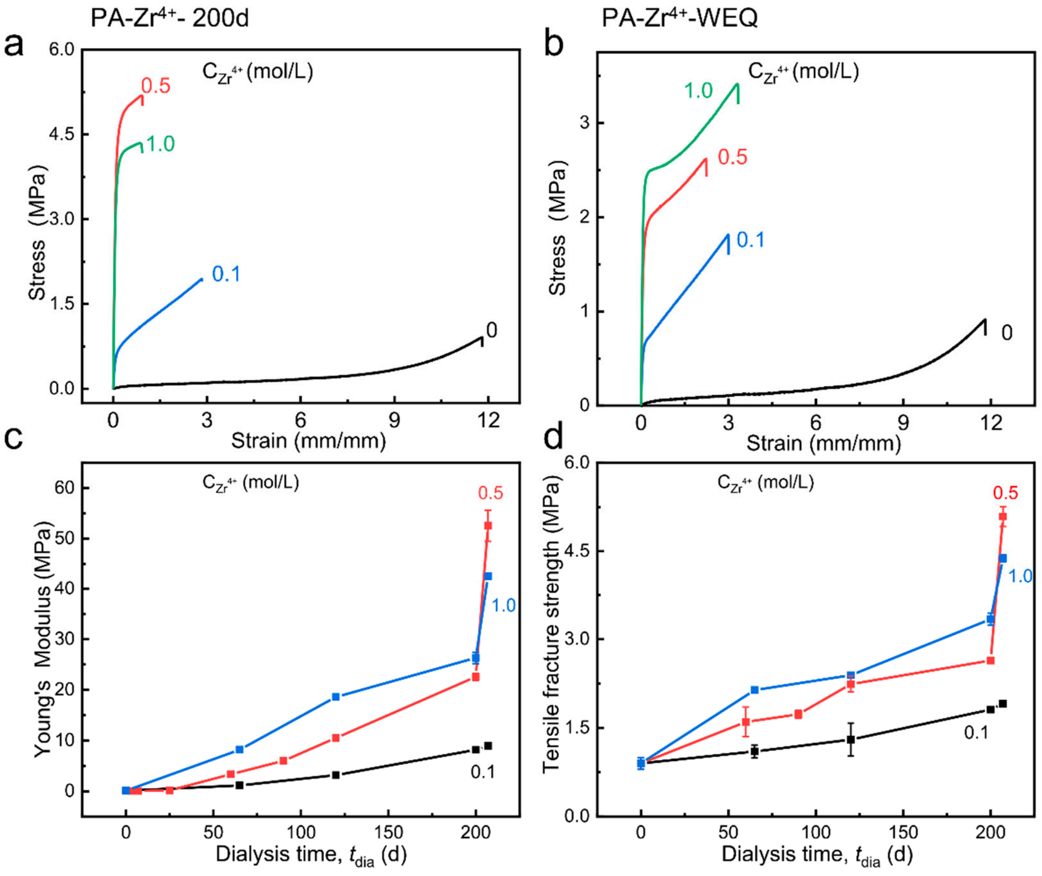

Figure S2. Tensile behaviors of PA-Zr^{4+} hydrogels equilibrated in different $ZrOCl_2$ solutions after 200 d and re-equilibrated in deionized water. (**a,b**) Stress-strain curves of the samples. (**c,d**) Detailed tensile properties of the samples.

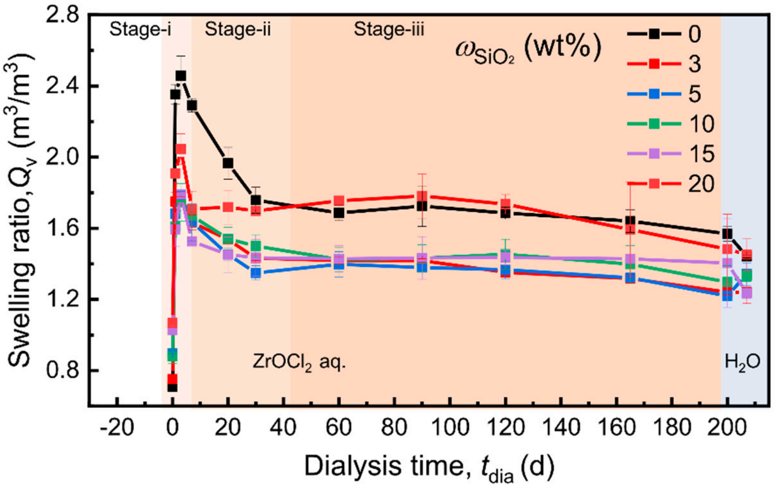

Figure S3. Swelling behavior of PA/SiO_2 nanocomposite hydrogel in 0.5 mol/L $ZrOCl_2$ solution and deionized water for different dialysis time (t_{dia}).

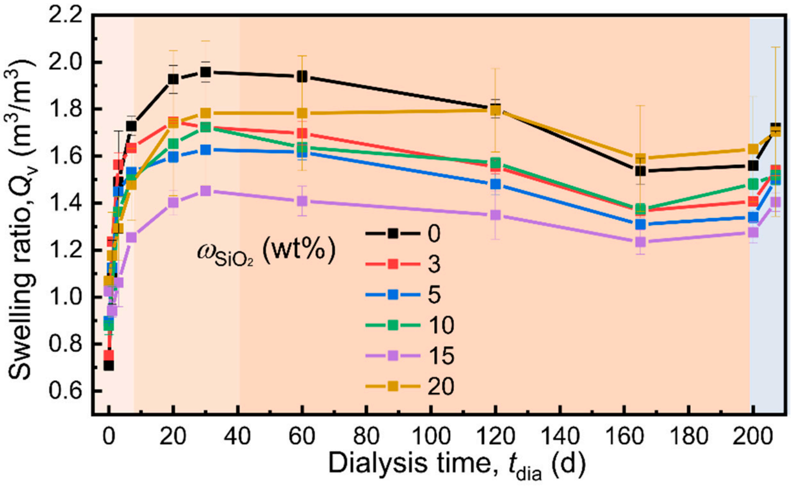

Figure S4. Swelling behavior of PA/SiO_2 nanocomposite hydrogel in 0.1 mol/L $ZrOCl_2$ solution and deionized water for different dialysis time (t_{dia}).

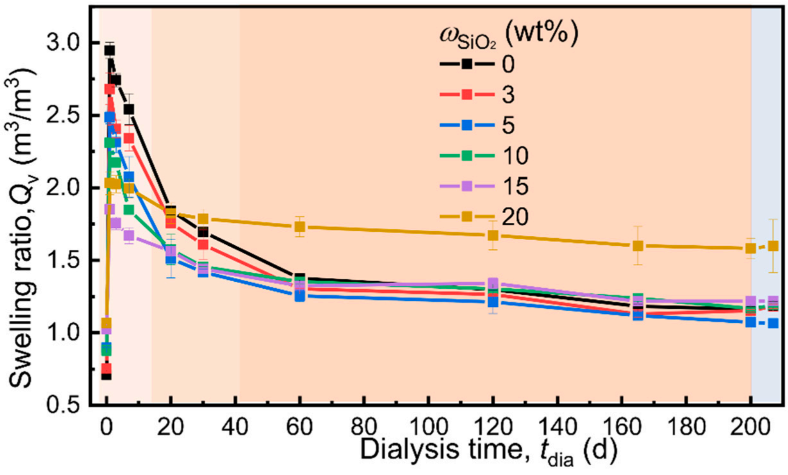

Figure S5. Swelling behavior of PA/SiO_2 nanocomposite hydrogel in 1.0 mol/L $ZrOCl_2$ solution and deionized water for different dialysis time (t_{dia}).

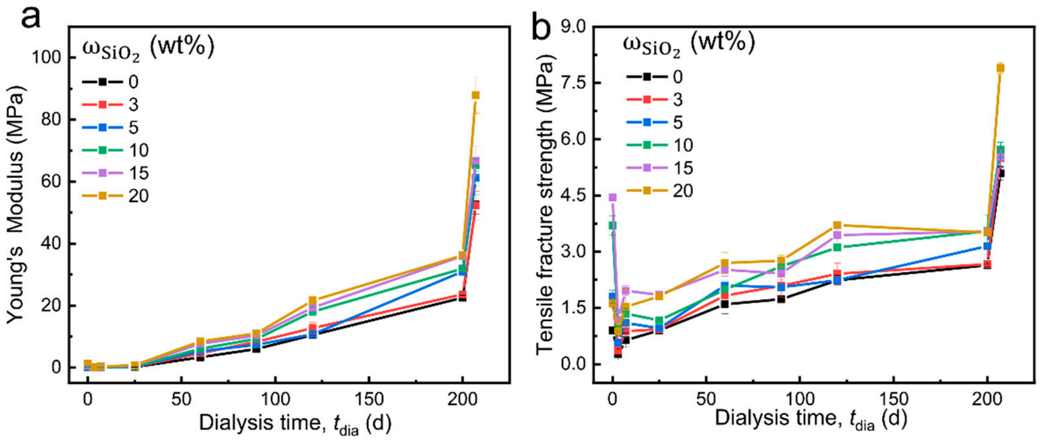

Figure S6. Tensile properties of PA/SiO_2 nanocomposite hydrogel in 0.5 mol/L $ZrOCl_2$ solution and deionized water for different dialysis time (t_{dia}). (**a**) Young's modulus, (**b**) Tensile fracture strength.

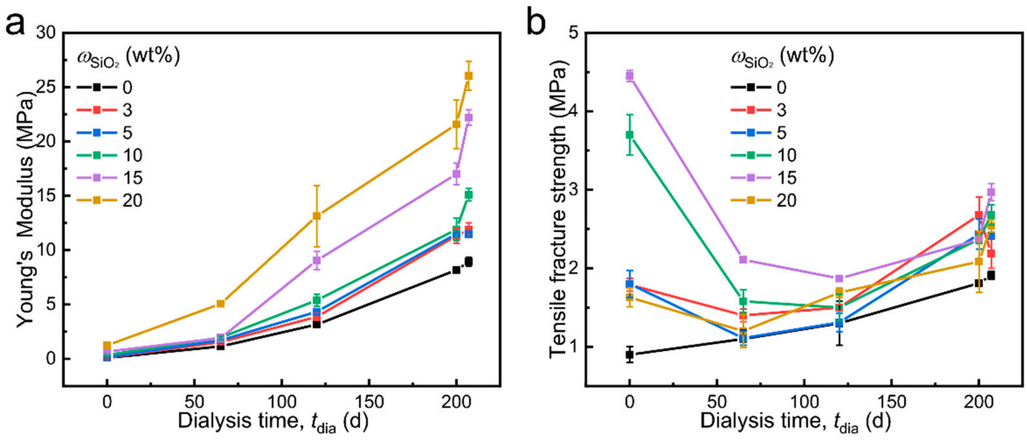

Figure S7. Tensile properties of PA/SiO_2 nanocomposite hydrogel in 0.1 mol/L $ZrOCl_2$ solution and deionized water for different dialysis time (t_{dia}). (**a**) Young's modulus, (**b**) Tensile fracture strength.

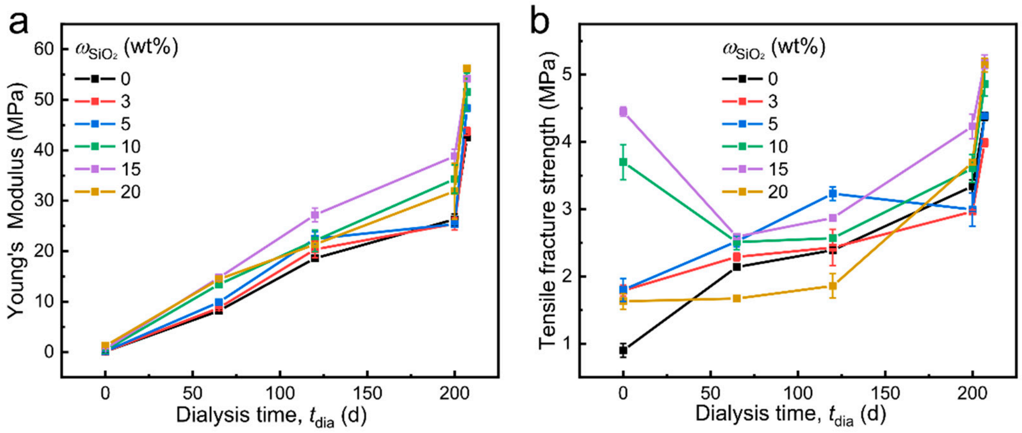

Figure S8. Tensile properties of PA/SiO_2 nanocomposite hydrogel in 1.0 mol/L $ZrOCl_2$ solution and deionized water for different dialysis time (t_{dia}). (**a**) Young's modulus, (**b**) Tensile fracture strength.

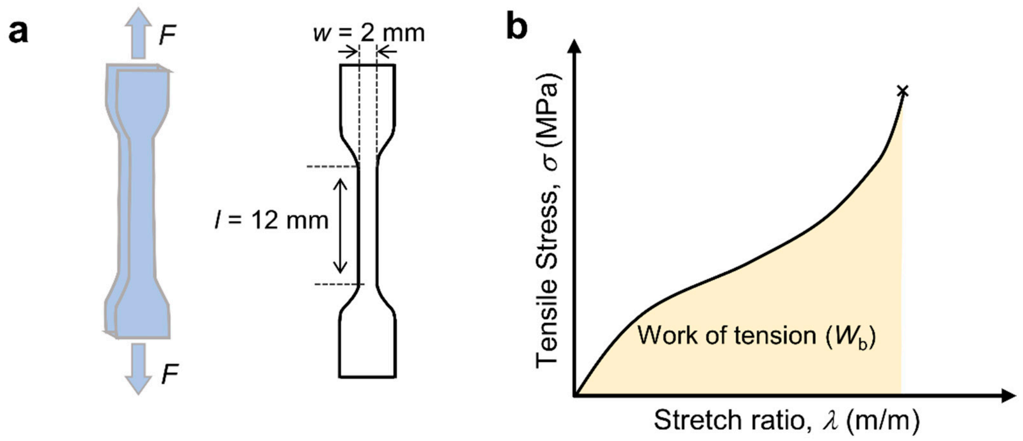

Figure S9. Sample geometry and method for tensile tests. (**a**) Geometry of tensile tests. The samples were cut into a dumbbell shape (standard: JIS-K6251-7) (thickness: t = 1–2 mm) prior to the tests. (**b**) Calculation of work of tension (W_b) by integrating the area under the stress-stretch curve.
